# Scale-Up of Early Infant Male Circumcision Services for HIV Prevention in Lesotho: A Review of Facilitating Factors and Challenges

**DOI:** 10.9745/GHSP-D-15-00231

**Published:** 2016-07-02

**Authors:** Virgile Kikaya, Rajab Kakaire, Elizabeth Thompson, Mareitumetse Ramokhele, Tigistu Adamu, Kelly Curran, Emmanuel Njeuhmeli

**Affiliations:** a Jhpiego/Lesotho, Maseru, Lesotho; b Jhpiego, Baltimore, MD, USA; c United States Agency for International Development, Division of Global HIV/AIDS, Washington, DC, USA

## Abstract

**Background:**

The World Health Organization and the Joint United Nations Programme on HIV/AIDS recommend early infant male circumcision (EIMC) as a component of male circumcision programs in countries with high HIV prevalence and low circumcision rates. Lesotho began incorporating EIMC into routine maternal, newborn, and child health (MNCH) services in 2013 with funding from the United States Agency for International Development and United Nations Children’s Fund. This presented unique challenges: Lesotho had no previous experience with EIMC and cultural traditions link removal of the foreskin to rites of passage. This process evaluation provides an overview of EIMC implementation.

**Methodology:**

The Lesotho Ministry of Health and Jhpiego conducted a baseline assessment before service implementation. Baseline information from an initial assessment was used to develop and implement an EIMC program that had a pilot and a scale-up phase. Key program activities such as staff training, quality assurance, and demand creation were included at the program design phase. Facilitating factors and challenges were identified from a review of information collected during the baseline assessment as well as the pilot.

**Results:**

Between September 2013 and March 2015, 592 infants were circumcised at 9 sites: 165 (28%) between 1 day and 6 days after birth; 196 (33%) between 7 and 30 days, and 231 (39%) between 31 and 60 days. Facilitating factors included strong support from the Ministry of Health, collaboration with stakeholders, and donor funding. Providers were enthusiastic about the opportunity to offer new services and receive training. Challenges included gaining consent from family members other than mothers, and parents’ concern about pain and complications. The EIMC program also had to manage providers’ expectations of compensation because overtime was paid to providers who took part in adult circumcision programming but not for EIMC. Limited human resources, including authorization only for doctors to perform EIMC, impeded provision of services.

**Conclusion:**

Despite communication, compensation, and task-shifting challenges, integrating EIMC services with MNCH services could be a sustainable model for EIMC service delivery in Lesotho.

## Abstract (precis)

Key elements of Lesotho’s phased introduction of early infant male circumcision were strong commitment from the Ministry of Health and donors; adequate training and supervision; integration with maternal, newborn, and child health; and appropriate communication. Challenges around cultural acceptance, the availability of health care providers, and task sharing will need to be addressed.

## INTRODUCTION

Observational and ecological studies indicated that male circumcision provides partial protection for men against HIV infection.^1^ Three randomized clinical trials confirmed that male circumcision reduces female-to-male HIV transmission by approximately 60%,^1^^-^^4^ and modeling studies^5^ showed that male circumcision indirectly reduces infections in women.^6^ Based on these findings, in 2007 the World Health Organization (WHO) and the Joint United Nations Programme on HIV/AIDS (UNAIDS) issued guidance urging countries with high HIV prevalence and low male circumcision rates to incorporate voluntary medical male circumcision (VMMC) into their HIV prevention programs.^7^ The guidance also recommended that “countries should consider how to promote neonatal circumcision in a safe, culturally acceptable and sustainable manner.”^8^

In response, the Lesotho Ministry of Health (MOH) created a male circumcision task force in 2007 and began implementation of adult VMMC services in February 2012 in partnership with the Maternal and Child Health Integrated Program (MCHIP), led by Jhpiego with financial support from the United States Agency for International Development (USAID)/U.S. President’s Emergency Plan for AIDS Relief (PEPFAR), and the United Nations Children’s Fund (UNICEF).^9^ Based on the success of the VMMC program, especially among younger boys, in 2013 the MOH piloted early infant male circumcision (EIMC) services as a component of its broader HIV prevention strategy to institutionalize male circumcision and reduce the need for future adult male circumcisions.^10^ These services were to be implemented not as a stand-alone program, like the VMMC program, but integrated with maternal, newborn, and child health (MNCH) activities.^11^ (See Box 1 for an implementation timeline.) This review describes the pilot and initial scale-up phase of the EIMC program in Lesotho.

**BOX 1. Timeline for Implementation of the Early Infant Male Circumcision Program (EIMC) in Lesotho**

- 2007: Male circumcision task force formed
- February 2012: Ministry of Health (MOH) launches voluntary medical male circumcision (VMMC) services
- February 2013: MOH commits to introduction of EIMC services
- April and May 2013: Rapid assessment of initial facilities
- September 2013: 4-month pilot launched
- February 2014: Start of phased scale-up

## BACKGROUND

### Early Infant Male Circumcision

As per WHO guidance, VMMC programs need adult circumcision to reach 80% coverage and EIMC to be institutionalized to maintain the benefit of the fast scale-up of the adult circumcision program.^12^ Compared with VMMC, the EIMC procedure is less complicated because it uses clamp devices that do not require stitches, healing is faster, and the complication rate is lower.^13^ EIMC also avoids barriers faced by adolescents and adults, including fear of pain, fear of HIV testing and learning one’s serostatus, and indirect costs—such as lost days of work or school for healing or return visits.^9^^,^^14^ In addition, EIMC is performed before initiation of sexual activity so there are no concerns about pain from erections or resumption of sexual activity before the wound heals.^13^^,^^15^ EIMC can be offered at a lower cost than VMMC and implemented economically in developing countries hard hit by HIV/AIDS. One study in 2010 estimated the cost of neonatal male circumcision at US$15 and adolescent and adult male circumcision at US$59.^16^ Thus, some international health experts have recommended that countries consider the provision of EIMC services.^16^ Nonetheless, EIMC cannot be considered as the sole VMMC service for HIV prevention because the reduction of HIV incidence through EIMC will take decades.

According to WHO guidelines, EIMC is safe for full-term infants who weigh more than 2.5 kg and who have the procedure from 12 to 24 hours through 60 days after birth under local anesthesia and with the use of WHO-recommended devices.^13^ While VMMC programs are usually stand-alone programs, EIMC can be integrated into existing, routine MNCH programs, including immunization clinics.^17^^,^^18^

EIMC projects have faced challenges around uptake of services in East and Southern Africa, where, unlike in West Africa, infant male circumcision is not commonly practiced.^17^ For example, in some communities of Botswana and Zimbabwe, infants are considered too young for circumcision because male circumcision is linked with rites of passage to adulthood^16^^,^^19^ and newborns are thought to be too vulnerable for surgery.^19^ Another challenge is obtaining consent: in most settings, only 1 parent is required to provide written consent for EIMC, but many new mothers prefer to involve other family members in the decision, including fathers and grandparents. Yet, these family members are often not present to provide consent when the mother has delivered or goes for postnatal care.^20^^,^^21^

On the service side, in most countries in East and Southern Africa, only physicians are licensed to provide male circumcision, while nurse-midwives and nonphysician clinicians are the primary care providers during and after delivery.^15^ In some countries, including Lesotho and Zimbabwe, concerns have been raised about the feasibility of introducing EIMC services into health services that are already overburdened and short-staffed.^22^ Despite these challenges, countries in the region have an opportunity to tackle HIV prevention and address broader neonatal and maternal health issues by scaling up EIMC.

### Lesotho Context

Lesotho has one of the highest HIV prevalence rates in the world, with an estimated 23.7% of adults infected.^23^ The national HIV prevention strategy identifies VMMC as a priority component of the national response.^24^ The Lesotho VMMC implementation plan, developed in 2012, outlines the 5-year strategy for reaching 80% of eligible males ages 15 to 49 years and institutionalizing EIMC services.^25^ Modeling studies have estimated that for every 5 circumcisions in Lesotho, 1 HIV infection would be prevented.^6^^,^^26^ VMMC services were introduced in Lesotho in 2012 at 4 fixed sites. The MOH organized scale-up at the national level and integrated VMMC services at all hospitals before initiating services at health centers through regular outreach. The MOH decided to introduce EIMC services as soon as the adult VMMC program was launched. In Lesotho, services are offered free of charge, but only doctors can perform the procedure, which is a challenge because of the limited number of health care providers in Lesotho.

Modeling studies have estimated that for every 5 circumcisions in Lesotho, 1 HIV infection would be prevented.

In 2013, the MOH established an EIMC task force within the Family Health Division, which oversees MNCH activities. The MOH held meetings to present the strategy and to determine the best approach to introduce EIMC in Lesotho. Meetings brought together a number of experts from United Nations (UN) agencies and other organizations supporting the MOH in the implementation of core HIV prevention and treatment programs. Stakeholders included MNCH, sexual and reproductive health, and family planning technical leads in the MOH and members of district management teams, UN agencies, the Christian Health Association of Lesotho, international and local NGOs, and managers at the selected hospitals. These meetings were organized to discuss guidelines for EIMC services in Lesotho and to determine how provision of EIMC services could be used as an opportunity to strengthen other MNCH services.

**Figure:**
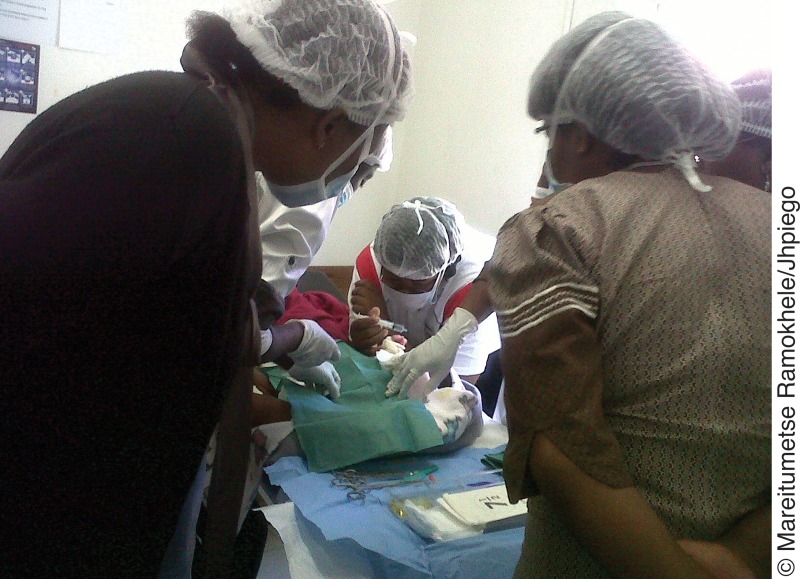
Health care providers receive on‐site training in early infant male circumcision at a district hospital in Lesotho.

The proposed program model sought to integrate EIMC services into existing MNCH services, with EIMC provided to infant boys between 1 and 60 days after birth. The stakeholders developed a road map for implementation of EIMC services that included the comprehensive package of services recommended by UNAIDS (Box 2).^12^

The proposed program model sought to integrate EIMC services into existing MNCH services, with EIMC provided to infant boys between 1 and 60 days after birth.

**BOX 2. Early Infant Male Circumcision (EIMC) Proposed Package of Services**

- Information about EIMC for parents
- Processes to document HIV status of the mother; if not known, HIV testing and counseling
- Adherence counseling for mothers in the prevention of mother-to-child transmission of HIV program to continue with their medication
- Counseling and consent from mother or parents
- Infant feeding education
- Messaging to reinforce postnatal visit on day 7
- Information to reinforce immunization
- Aftercare instructions—post-EIMC care
- Analgesics
- General HIV prevention pamphlets
- Condom provision to mothers

Based on programs in other countries, and after discussion and consultation with the EIMC task force, the Lesotho MOH chose the Mogen clamp for program scale-up, because it is a 1-piece device, it does not need to be assembled, and it can be reused after sterilization. Other devices under consideration are placed on the infants and require a return visit to the facility for removal, which is a challenge due to Lesotho’s mountainous terrain.

Both doctors and nurses were trained on the EIMC procedure with specific roles. The procedure is performed only at the hospital level and only by doctors; however, trained nurses assist with the surgical procedure and perform follow-up care of circumcised infants. In Lesotho, either parent of the male infant can provide written consent for the procedure even though culturally fathers are expected to be the ones who authorize mothers to circumcise the infant.

## METHODS

A pilot of EIMC services in Lesotho was conducted over 2 years from September 2013 to March 2015. A phased approach was used to ensure that all steps of program implementation were in place. Three phases were designed: a rapid assessment to gather baseline information regarding readiness of facilities as well as potential demand (see Supplementary Material 1: Facility readiness assessment form); a pilot phase at 2 facilities; and a moderate program scale-up phase with services being rolled out at 6 other hospitals and 1 health center. Members of the EIMC task force assessed each phase and drew up recommendations to inform the following phase.

Data collected included numbers of EIMCs provided and results from the rapid assessment, which included information collected from interviews of key informants and stakeholders. The EIMC task force held discussions to identify potential facilitating factors and challenges to the scale-up of the national EIMC program. At the 2 facilities, the MOH and Jhpiego conducted an initial training for providers (doctors and nurses) after their selection by hospital management. The program procured equipment and supplied it at each site (e.g., Mogen clamps and restraining boards). Nurses conducted education on EIMC at antenatal care (ANC) clinics at hospitals and health centers. Circumcised babies were routinely assessed at 48 hours and 7 days after the procedure, either in the hospital or at their health center, to evaluate wound healing and to detect and document adverse events.

### Ethical Considerations

Ethical oversight was provided by the Lesotho MOH. Data presented in this manuscript are anonymous and analysis was done without identifiers. Informed consent to circumcise the child was obtained from the parent after information highlighting benefits and risks of the procedure was given. The local institutional review board (IRB) and the Johns Hopkins University IRB provided approval for secondary data analysis.

## RESULTS

### EIMC Provision

During the pilot, 45 providers, including 4 doctors, 25 hospital nurses, and 16 health center nurses were trained during 3 workshops to perform various aspects of EIMC services. During the scale-up phase, 149 health workers, comprising 131 nurses, 16 doctors, and 2 lay counselors, received training in 9 workshops at the 7 sites where services were scaled up (Table 1).

**TABLE 1:** Number and Cadre of Providers Trained in Lesotho From September 2013 to March 2015

	Pilot (Sep 2013–Jan 2014)	Scale-Up (Feb 2014–Mar 2015)	Total
Number of training workshops	3	9	12
Total number of providers trained	45	149	194
Doctors	4	16	20
Nurses at hospitals	25	60	85
Nurses at health centers	16	71	87
Lay counselors	–	2	2

Between the start of the pilot in September 2013 and March 2015, 592 male infants were circumcised (Table 2), including 40 infants circumcised during the pilot at Mafeteng District Hospital and Scott Hospital. A total of 28% of infants were circumcised at 1 to 6 days after birth, 33% at 7 to 30 days after birth, and 39% at 31 to 60 days after birth. Most infants were circumcised after the neonatal period; no infants were circumcised from 12 to 24 hours after birth.

**TABLE 2:** Number and Timing of EIMCs and Adverse Events in Lesotho From September 2013 to March 2015

	Pilot Phase (Sep 2013–Jan 2014)	Scale-Up (Feb 2014–Mar 2015)	Total, No. (%)
Total number of infants circumcised	40	552	592 (100%)
Age at circumcision, days			
1–6	5	160	165 (28%)
7–30	14	182	196 (33%)
31–60	21	210	231 (39%)
Adverse events	0	1	1 (0.2%)

Abbreviation: EIMC, early infant male circumcision.

Only 1 mild adverse event was recorded during the reporting period, representing 0.2% of total circumcisions. No severe adverse events were recorded. The adverse event occurred on a procedure performed during a training activity by a trainee doctor on a 6-week-old infant at Mafeteng District Hospital. Upon completion of the procedure, it was noted that excessive skin had been removed from the shaft on the ventral aspect of the penis. The trainer on-site consulted with the master trainer off-site and a decision was made to allow for healing to proceed and to determine any remedial action at the infant’s follow-up visit after 48 hours. At the 48-hour visit, the wound was exposed and assessed. The infant was examined again at the seventh day post-circumcision and again at 4 weeks post-circumcision. By the third checkup and review visit, the wound was satisfactorily healed and the defect had been covered by new skin.

From October 2014 through March 2015, 11% (246/2,171) of male babies delivered at the 9 implementation sites (2 hospitals of the pilot, 6 hospitals and 1 health center of the scale-up) were circumcised; rates ranged from 27% (81/299) in Berea Hospital to 2% (8/403) in Leribe Hospital.

### Initial Rapid Assessment of Pilot Facilities

The rapid assessment in May 2013 provided information for upgrading sites to offer EIMC services. Specifically, it assessed MNCH programs in selected facilities, identifying opportunities and mechanisms for integrating EIMC services with MNCH services, sensitizing site managers and health care providers on the introduction of EIMC services in their facilities, and assessing the willingness of parents expecting a baby, should they have a son, to allow him to be circumcised.

The methodology for data collection included interviews with site managers, health care providers, and expectant parents (and accompanying relatives) attending ANC services, data collection of key information (including statistics regarding deliveries of male babies, opportunities for demand creation, range of MNCH services provided, human resource capacity, infection prevention and control practices, and proposed space for EIMC services), and direct observation of newborn care services (see Supplementary Material 2: Interview questionnaire for potential parents).

The rapid assessment was conducted at 2 sites that were considered for the pilot phase. These sites were selected as they were already offering VMMC (the doctors at those sites were trained for adult VMMC provision), and they were close to the MOH headquarters in the capital—for ease of monitoring. The 2 sites were Mafeteng District Hospital and Scott Hospital (see Supplementary Material 3: Feasibility facility assessment).

The findings from the assessment showed that both hospitals’ proposed locations for EIMC required improvements for safe provision of services and that 2 locations for EIMC would be needed in both hospitals: one at the maternity ward and the other at the postnatal care clinic. All staff interviewed at the hospitals expressed an interest in the introduction of EIMC services in their respective facilities and a willingness to be trained. Most of the interviewees (expectant parents) at the hospital also showed an interest in EIMC services. The parents raised concerns about pain for the neonate, timing of the surgery, and potential negative consequences, such as mutilation of the newborn by the EIMC procedure. However, a majority of those interviewed thought that EIMC was good for health and reduced chances of infections.^9^ The interviews indicated that a communication plan would need to address community concerns around EIMC (Box 3).

**BOX 3. Recommendations From the Rapid Assessment for the Pilot**

- Establish an early infant male circumcision (EIMC) task team to oversee introduction of EIMC services in hospitals
- Develop guidelines and tools for service provision and monitoring
- Continue discussions around task sharing to allow nurses to perform EIMC procedures at both hospitals and clinics
- Implement EIMC services where feasible on a daily basis, not on a weekly basis (voluntary medical male circumcision services are currently provided twice a week)
- Develop EIMC information, education, and communication materials for communities

### Pilot at 2 Facilities

EIMC services were introduced at the 2 pilot facilities in September 2013. The objectives of the pilot were to introduce EIMC services at the 2 hospitals and to consider integration of EIMC services into MNCH services. Specific objectives included developing appropriate trainings for the Lesotho context; finalizing and implementing EIMC program guidelines; and ensuring targeted communication on EIMC services through the development and distribution of information, education, and communication (IEC) materials; as well as education of pregnant women. Key documents developed during this phase included guideline documents, “Minimum Standards for Performing Early Infant Male Circumcision” (see Supplementary Material 4), a group education and counseling flipchart, a step-wise procedure chart to guide health workers in performing circumcision with the Mogen clamp, a complications pamphlet, and IEC brochures (See Supplementary Material 5a, b, c: IEC materials). Monitoring and evaluation tools, including registers, adverse event forms, and quality standards assessment tools, were also developed and piloted at the 2 hospitals. When finalizing EIMC guidelines, it became clear that there was a need also to finalize and update guidelines on essential newborn care.

Results from the pilot showed that, for the most part, providers felt comfortable with their level of training and capable of mobilizing and educating families and assisting with or performing the procedure. Provider concerns primarily revolved around limited staffing and time allotted to EIMC services in the context of insufficient human resources at hospitals (Box 4).

**BOX 4. Recommendations From the Pilot for the Scale-Up**

- Continue task-sharing discussions
- Explore ways to increase male involvement to improve rates of facility births, infant care, and overall child health
- Scale up demand creation for services, including during antenatal care, by increasing awareness of early infant male circumcision (EIMC) availability
- Train additional providers from existing and new service sites to ensure the expansion of services
- Ensure the quality of services at all sites by providing training for all managers and health care workers involved in service provision
- Ensure that the few trained staff are scheduled to provide services daily to avoid missed opportunities due to staff unavailability

### Scale-Up at 7 Sites

A roll-out plan detailing steps to be taken to expand services nationally was developed with consideration of recommendations from the pilot (Box 5). EIMC services were started at an additional 7 sites between February 2014 and March 2015. These included Carewell Clinic and St. Joseph’s Hospital in Maseru district; Berea Hospital and Maluti Adventist in Berea district; Motebang Hospital in Leribe district; Butha-Buthe District Hospital; and Ntsekhe Hospital in Mohale’s Hoek district.

**BOX 5. Key Steps to Introduce Early Infant Male Circumcision Services During National Scale-Up**

- Engage facility management team
- Conduct rapid site assessment—look at infrastructure and personnel
- Conduct initial demand creation in hospital and community
- Set up site
- Select and train providers
- Initiate services
- Provide supportive supervision and mentoring

## KEY PROGRAM ACTIVITIES

### Training of EIMC Service Providers

Training for both the pilot and scale-up phases took place immediately before service initiation at each of the implementing sites. Eligible trainees were qualified and licensed health care workers, preferably working in the maternity unit or in the maternal and child health clinic at the hospitals where service initiation was being planned. Each training ran for 5 days, and doctors and nurses trained together. Trainers conducted regular visits and worked with EIMC trainees until they reached competency in the circumcision procedure and could identify and manage complications. Other components of the training included education and counseling for parents of male infants. Specific training was developed for nurses at health centers on providing education and counseling of prospective parents, pre-procedure screening to ensure infants were fit for circumcision, and post-procedure checkup and care.

Each training ran for 5 days, and doctors and nurses trained together.

Teaching methods included 1 day in the classroom for theory presentations and discussion, 1 day of practice with models at different skills stations, and 3 days of hands-on clinical practice with babies in the clinic. To ensure competence, trainers closely monitored and supervised the physicians and nurses during the first 5 procedures. Trainers assessed knowledge at the end of the training through an examination, and they assessed skills by observing each step of the procedure.

### Demand Creation and Social Mobilization for EIMC

The EIMC technical working group developed a communication plan to address myths and misconceptions about EIMC. IEC materials regarding EIMC services are continuing to be provided to the primary audience (parents and potential parents of male infants) at ANC clinics, maternity wards, postnatal clinics, clinics seeing children under the age of 5, and VMMC clinics. These IEC materials were also provided through routine community mobilization activities.

### Quality Assurance

Early in program implementation, the technical working group developed tools for quality assurance. The MOH, WHO, and Jhpiego conducted external quality assurance visits to all sites. Lesotho uses guidelines based on WHO’s quality assurance guidelines for male circumcision.

In addition, each site has a quality assurance assessment team (MOH technical staff, site staff, and EIMC providers from a different site) that undertakes a quality assurance review, to identify performance gaps and to develop action plans to bridge those gaps. EIMC service providers and managers also conduct periodic self-assessments of their services to improve quality. The process helps providers develop a sense of ownership of the assessment findings so that they become involved in making recommendations and implementing solutions.

## DISCUSSION

While the EIMC program in Lesotho is still in the early stages, demand for services has increased slowly as the program has expanded from 2 to 9 sites.^9^

Introduction of the EIMC program was supported by a strong commitment from the MOH, close cooperation with stakeholders (UNICEF, USAID, the Christian Health Association of Lesotho, etc.), and stable funding from USAID and UNICEF. In addition, when providers were offered the chance to train, they were enthusiastic about the opportunity to acquire a new skill and offer new services. The phased introduction of services helped to ensure a smooth start-up and, where possible, recommendations from the assessment were applied to the pilot, and recommendations from the pilot were applied to the scale-up at 7 facilities (see Supplementary Material 6: EIMC pilot implementation program assessment).

With the launch of EIMC and its integration with the MNCH program, it became apparent that the national neonatal care manual had not been finalized. As a result, the MOH updated and finalized the manual, which now includes an EIMC component. Providers being trained in EIMC now receive refresher training on neonatal care, which is important because of Lesotho’s high infant mortality rate (74 deaths per 1,000 live births).^21^ The program will continue to review the EIMC service provision package for potential improvements. The program is also exploring the possibility of using the provision of EIMC services as an opportunity to increase male involvement in infant care, increase facility-based births, and improve infant health care generally.

Providers being trained in EIMC now receive refresher training on neonatal care, which is important because of Lesotho’s high infant mortality rate.

### Challenges

The primary challenge to EIMC uptake in Lesotho is that even though either parent can provide consent individually, the mother still consults with the father and other family members before consenting to the procedure, as has been reported in other programs in Southern Africa.^19^^,^^27^ In Lesotho, a mother can provide legal consent for EIMC, but most prefer to consult with the father as well as other family members about the decision to have a male child circumcised, which can delay the procedure. In Lesotho, most fathers do not accompany mothers to ANC visits, to the hospital for the birth, or to postnatal care visits, the main sources of EIMC information. Therefore, most fathers are reluctant to give consent since they do not have firsthand information about the safety and benefits of the procedure. Possible solutions to this challenge include ensuring that fathers are invited to the facility to give their informed consent and providing informed consent forms to the mother during ANC visits in order to get the father’s consent prior the baby’s birth.

The primary challenge to EIMC uptake in Lesotho is the mother’s need to consult with the father and other family members before consenting to the procedure.

In Lesotho, low rates of institutional childbirth (58.7%)^28^ and limited use of neonatal and early infant care services also limit access to EIMC information and services as many infants’ first contact with the health system is at their 6-week immunization visit.^29^ Early discharge from the hospital also limits access to EIMC. As per the Lesotho guidelines, mothers who deliver normally at hospitals are discharged as early as 4 to 6 hours postpartum, which limits opportunities to provide EIMC after the recommended 12 hours.

Cultural practices also have an impact on uptake of EIMC services. Traditionally, boys in Lesotho are circumcised during ritual initiation into adolescence and adulthood.^28^ Some mothers feel that infancy is not the proper time to have their sons circumcised because they fear their sons will not be accepted for initiation.^9^ Research is needed to determine how best to scale up and tailor demand creation activities to address the Lesotho context.

Another challenge in Lesotho and supported by other studies is parents’ reluctance to have their baby boys circumcised due to concerns about pain, the infant being too young to undergo a surgical procedure, and potential harm.^15^^,^^20^ The Lesotho VMMC/EIMC program addressed these concerns through its IEC materials and expects that as more babies are circumcised, the very low rate of adverse events will help allay these concerns.

The program also had some difficulties integrating EIMC services into the MNCH program. The VMMC program in Lesotho is a stand-alone program that operates year-round from clinics and through seasonal campaigns. While MNCH providers have been enthusiastic about getting trained in EIMC service provision, this enthusiasm has been hard to sustain, one reason being compensation. Unlike VMMC providers, EIMC providers do not receive incentives for providing male circumcision services. The MOH sees the provision of EIMC services as part of the standard package of MNCH services and has not provided for extra compensation. As a result of the discrepancy, some MNCH providers have lost enthusiasm for including additional tasks in their already busy schedules. In addition, some hospitals that provided EIMC services for a fee before the launch of the official EIMC program are now expected to provide these services for free, which has undermined management buy-in.

A notable challenge is the lack of available providers, which is partly the result of a shortage of doctors, the only cadre authorized by their regulatory body to perform EIMC. However, under the new, recently revised preservice education curriculum, nurses are being trained to perform the EIMC procedure. In practice, most nurse clinicians are not sure they have the authority from their regulatory body and are hesitant to perform the procedure. Furthermore, nurse clinicians are a recently revived cadre and very few in number, and therefore they cannot meet the demand for EIMC services. There are fewer than 50 nurse clinicians for the entire country. All cadres of nurses should be authorized to perform EIMC to meet the growing demand. The need for task sharing of the male circumcision surgical procedures between medical doctors and nurses was identified before program inception and is seen in other programs as well.^30^ This need is especially important in Lesotho where, due to the deficit of medical doctors, maternity units are usually not staffed with full-time doctors. Doctors assigned to maternity units are often available only for emergencies as they usually do not have time for providing routine services. We have seen that there are times in hospitals when nurses, who are the primary providers of information on EIMC, have women who would like to have their babies circumcised, but a doctor is not available. In addition, EIMC is supposed to be routinely performed in maternity units and postnatal clinics, which are frequently staffed with nurses who are currently not allowed by their regulatory organization to perform the procedure. Furthermore, most mothers who deliver at hospitals are encouraged to go to health centers for their postnatal clinic checkups, where EIMC is presently not offered. Studies in East and Southern Africa have shown that nurses and clinical officers can be trained to provide EIMC safely.^4^^,^^31^

## CONCLUSION

The introduction of EIMC services for HIV prevention into MNCH services in Lesotho demonstrates the feasibility of such a program in a low-resource setting with leadership and commitment from the MOH and key stakeholders. EIMC can be institutionalized to complement gains from the adult VMMC program.

Allowing nurses to perform EIMC procedures would increase the availability of services in hospitals and would also allow the procedures to be performed in health centers, where a large number of potentially eligible infants are seen. Stakeholders should encourage the MOH and professional regulatory organizations to license nurses to provide EIMC services.

Circumcision services should also be offered on a daily basis at implementation sites. Currently, EIMC services are offered once or twice a week at each site, based on the providers’ workload or on set service days. We also believe that the MOH should explore offering EIMC services on a daily basis at district hospitals. EIMC services would then be available during the 3 visits (delivery, postnatal care Day 7, and postnatal care Week 6) that are most likely to present an opportunity for babies to be circumcised, and the mothers would not have to make a return visit, which is difficult for them.

With only 1 adverse event reported during the assessment, we believe that this review will assist program implementers in Lesotho to tailor the national scale-up of services to their local context.

## Notes

**Peer Reviewed**
